# Research on a Support-Free Five-Degree-of-Freedom Additive Manufacturing Method

**DOI:** 10.3390/mi15070855

**Published:** 2024-06-30

**Authors:** Xingguo Han, Gaofei Wu, Xuan Liu, Xiaohui Song, Lixiu Cui

**Affiliations:** 1 College of Mechanical and Control Engineering, Guilin University of Technology, Guilin 541006, China; hanxingguo2004@163.com; 2 Guangxi Key Laboratory of Special Engineering Equipment and Control, Guilin University of Aerospace Technology, Guilin 541004, China; sxhui@guat.edu.cn (X.S.); cuilixiu@guat.edu.cn (L.C.); 3 Key Laboratory of Special Engineering Equipment Design and Intelligent Driving Technology, Education Department of Guangxi Zhuang Autonomous Region, Guilin University of Aerospace Technology, Guilin 541004, China; 4 School of Mechanical and Electrical Engineering, Guilin University of Electronic Technology, Guilin 541004, China; 17863642183@163.com

**Keywords:** 3D printing, additive manufacturing, five-degree-of-freedom, support-free

## Abstract

When using traditional 3D printing equipment to manufacture overhang models, it is often necessary to generate support structures to assist in the printing of parts. The post-processing operation of removing the support structures after printing is time-consuming and wastes material. In order to solve the above problems, a support-free five-degree-of-freedom additive manufacturing (SFAM) method is proposed. Through the homogeneous coordinate transformation matrix, the forward and inverse kinematics equations of the five-degree-of-freedom additive manufacturing device (FAMD) are established, and the joint variables of each axis are solved to realize the five-axis linkage of the additive manufacturing (AM) device. In this research work, initially, the layered curve is obtained through the structural lines of the overhang model, and a continuous path planning of the infill area is performed on it, and further, the part printing experiments are conducted on the FAMD. Compared with the traditional three-axis additive manufacturing (TTAM) method, the SFAM method shortens the printing time by 23.58% and saves printing materials by 33.06%. The experimental results show that the SFAM method realizes the support-free printing of overhang models, which not only improves the accuracy of the parts but also the manufacturing efficiency of the parts.

## 1. Introduction

AM, also known as three-dimensional printing (3DP), rapid prototyping or solid freeform [1], originated in the 1980s [2]. It is a manufacturing method that deposits materials layer by layer to form parts with complex geometric shapes [3]. AM has the characteristics of high processing flexibility, so it is widely used in construction [4], biotechnology [5], medical [6], composite materials [7], military [8], aerospace [9] and other fields.

Currently, most AM fields use TTAM technology. Traditional 3D printing can only deposit materials along the positive *Z*-axis direction of the device, so it can actually only be called “2.5D” printing [3]. Since the printing material is affected by gravity, TTAM often requires printing the support structures at the same time as the outward-extending part of the model (i.e., the hanging part) in order to successfully print the entire part. However, adding support structures will increase the manufacturing time and reduce the manufacturing efficiency of the part. And the post-processing operation of removing the support part will not only increase the time cost but also affect the surface quality of the part. In order to solve a series of problems caused by the support structure, many support-free algorithms suitable for TTAM equipment have been proposed. Hu et al. [10] proposed an algorithm for approximate pyramid shape decomposition. They linked the pyramid decomposition problem to the exact coverage problem (ESP) and used the Knuth X algorithm to solve the ESP to achieve support-free printing. Herholz et al. [11] presented methods using a height field for the printability in a support-free manner. Wu et al. [12] developed a method to generate support-free infill structures on adaptive rhombic cells. Wei et al. [13] divided the model into the minimum number of parts for printing and then assembled them into final parts to achieve the effect of unsupported printing, but the assembly operation will lead to varying degrees of errors. Li et al. [14] proposed a support-free printing method that slices the solid model into a cluster of convex hull layers by using tri-variate T-splines and successfully printed the models of the upper and the lower extremity of the femur (which have both convex and concave structures) without introducing supporting structures. However, this method is not suitable for geometry with an arbitrary complex topology structure. Chen et al. [15] manufactured the internal core of the objects using universal building blocks and fabricated outer shells with pyramidal decomposition which can realize support-free printing, but their method does not explicitly consider imbalance, structural weaknesses or building strength.

In recent years, in order to reduce the impact of support structures during TTAM processes, many AM algorithms based on multiple degrees of freedom (larger than three degrees of freedom) have been proposed. Compared with TTAM equipment, AM equipment with extra degrees of freedom can achieve the support-free printing of parts by dynamically rotating the build axis, thereby improving part quality and shortening manufacturing time [16]. Shen et al. [17] designed a flexible platform composed of multiple sub-platforms. When printing overhanging models, the equipment can freely lift and lower each sub-platform and use it as a support surface for parts to achieve unsupported material printing. However, the existence of a bonded interface between the printed part and the sub-platform will affect the disassembly and surface accuracy of the part. Dai et al. [18] decomposed a model into curved surface layers so that the fabrication (such as bunny model) can be performed in a support-free way. The limitation of this method is that the genetic algorithm is computationally inefficient when the number of cutting planes is large. Gao et al. [19] combined multi-degree-of-freedom printing equipment with model decomposition methods to achieve printing with the lowest cost of supporting materials, but the computational efficiency of the cutting plane process is low. Wu et al. [20] presented a volume decomposition algorithm of planar clipping to realize support-free printing on either 4-DOF or 5-DOF systems, but the separate printed parts need to be assembled manually into a final model. Wang et al. [21] proposed a novel 5-axis dynamic slicing algorithm to achieve non-supporting material printing (NSMP). Liu et al. [22] proposed a new method of non-supporting thin-wall structure (NSTWS) manufacturing by using a multi-DOF robot arm, which can manufacture irregular thin-wall structure models such as elbow, a vase or a transition structure. In the view of the current volume decomposition planning method, Bi et al. [23] proposed an integrated strength–support volume decomposition optimization method that simultaneously achieves support-free printing and strength enhancement.

In this paper, we develop an SFAM method for parts with regular overhang structures to achieve support-free printing. In addition, a path planning method for continuous printing is proposed to reduce the idle stroke motion of the printing head and improve manufacturing efficiency. The structural line of the model is used to perform slicing on the model, and the deposition direction of the AM equipment is kept perpendicular to the part production layer to realize the support-free manufacturing of parts, so as to achieve the purpose of saving materials and improving manufacturing efficiency. Lee et al. [24] classified five-degree-of-freedom machining devices into three types: table-tilting type, spindle-tilting type and table/spindle-tilting type. The additional rotational motion in an FAMD of a table-tilting type is provided to the printing platform instead of the printing nozzle, and it can keep the gravitational acceleration of the nozzle and the printing consumable in the same direction, which helps in the deposition of materials when printing parts [25]. Therefore, the type of FAMD used in this paper is table-tilting. This study focuses on circular and rectangular cross-section models with overhang angles.

## 2. Working Principle of FAMD

**Figure 1:**
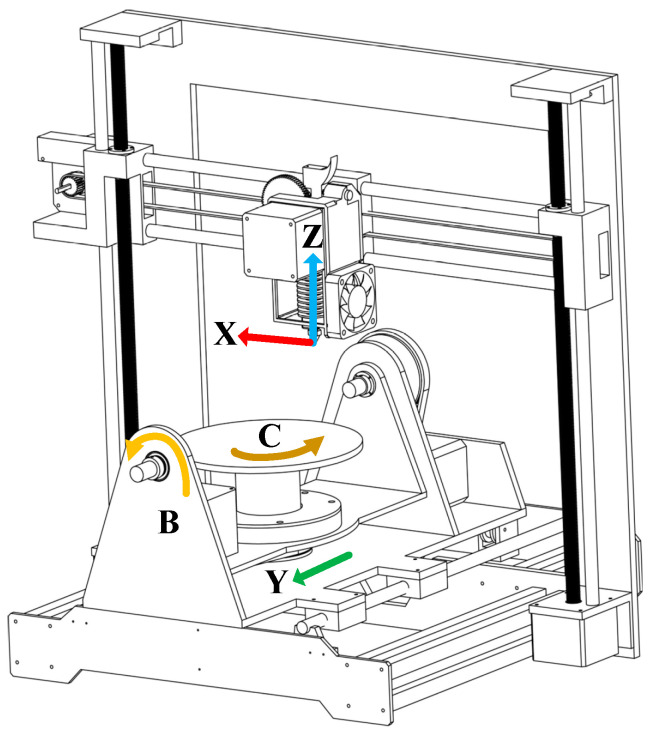
The model of the FAMD.

The model of the FAMD is shown in Figure 1. The left and right (horizontal) motion joint variables and the up and down (vertical direction) motion joint variables of the printing nozzle are the X value and Z value, respectively; the forward and backward motion joint variables of the printing platform are the Y value; the B value is the joint variable of the printing platform for flipping motion; the C value is the joint variable used by the printing platform to perform rotational motion. In order to achieve support-free printing with the FAMD, the angle of the printing table needs to be adjusted at all times when manufacturing parts. During the printing process, keeping the deposition direction of the printing material perpendicular to the plane of the pre-printed model can reduce errors caused by the step effect and achieve support-free printing. In order to obtain the tilt angle and rotation angle of the printing workbench, a kinematic analysis of the FAMD is essential.

### 2.1. Analysis and Solution of Forward Kinematics for FAMD

When the FAMD prints parts with overhanging features, the printing platform needs to be changed at a certain angle. In order to ensure that the equipment can correctly implement the unsupported printing of an FAMD, it is necessary to obtain the spatial posture relationship of the printing platform relative to the printing nozzle through a spatial posture transformation matrix.

**Figure 2:**
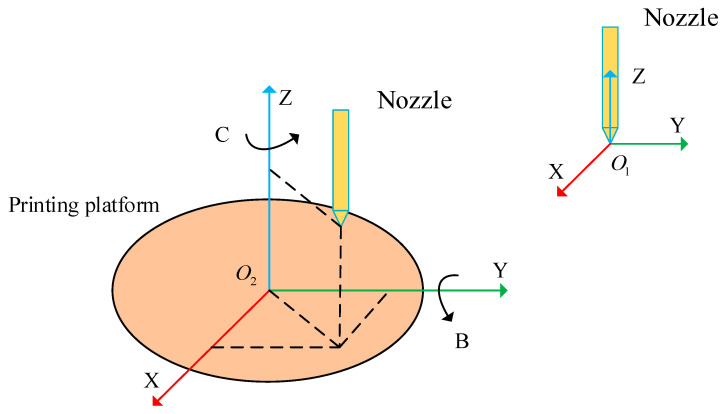
The forward kinematics analysis of the FAMD.

The spatial posture relationship of the printing nozzle and printing platform is shown in Figure 2. The attitude relationship between the printing nozzle and the printing platform can be represented by a rotation transformation matrix, as shown in Equation (1).

(1) $$Rot\left(CB\right)={Rot}_{Z}\left(C\right){Rot}_{Y}\left(B\right)$$

where ${Rot}_{Z}\left(C\right)$ is the transformation matrix rotating around the *Z*-axis with an angle C, as shown in Equation (2); ${Rot}_{Y}\left(B\right)$ is the transformation matrix rotating around the *Y*-axis with an angle B, as shown in Equation (3).

(2) $${Rot}_{z}\left(C\right)=\left[\begin{array}{cccc} cosC & -sinC & 0 & 0\\ sinC & cosC & 0 & 0\\ 0 & 0 & 1 & 0\\ 0 & 0 & 0 & 1 \end{array}\right]$$

(3) $${Rot}_{Y}\left(B\right)=\left[\begin{array}{cccc} cosB & 0 & sinB & 0\\ 0 & 1 & 0 & 0\\ -sinB & 0 & cosB & 0\\ 0 & 0 & 0 & 1 \end{array}\right]$$

The positional relationship between the printing nozzle and the printing platform can be expressed by the translation transformation matrix, as shown in Equation (4).

(4) $$Tran\left(X,Y,Z\right)=\left[\begin{array}{cccc} 1 & 0 & 0 & X\\ 0 & 1 & 0 & Y\\ 0 & 0 & 1 & Z\\ 0 & 0 & 0 & 1 \end{array}\right]$$

Through Equations (1) and (4), the pose relationship between the printing nozzle and the printing platform can be obtained, which can be represented by the transformation matrix T, as shown in Equation (5).

(5) $$T=Rot\left(CB\right)\;Tran\left(X,Y,Z\right)$$

Substituting Equations (1)~(4) into Equation (5), the transformation matrix T can be solved, and the result is shown in Equation (6).

(6) $$T=\left[\begin{array}{cccc} cosBcosC & -sinC & sinBcosC & XcosBcosC-YsinC+ZsinBcosC\\ cosBsinC & cosC & sinBsinC & XcosBsinC+YcosC+ZsinBsinC\\ -sinB & 0 & cosB & ZcosB-XsinB\\ 0 & 0 & 0 & 1 \end{array}\right]$$

The direction vector of the printing nozzle can be obtained through Equation (6), as shown in Equation (7).

(7) $$\left[\begin{array}{c} i\\ j\\ k \end{array}\right]=\left[\begin{array}{c} sinBcosC\\ sinBsinC\\ cosB \end{array}\right]$$

### 2.2. Analysis and Solution of Inverse Kinematics for FAMD

**Figure 3:**
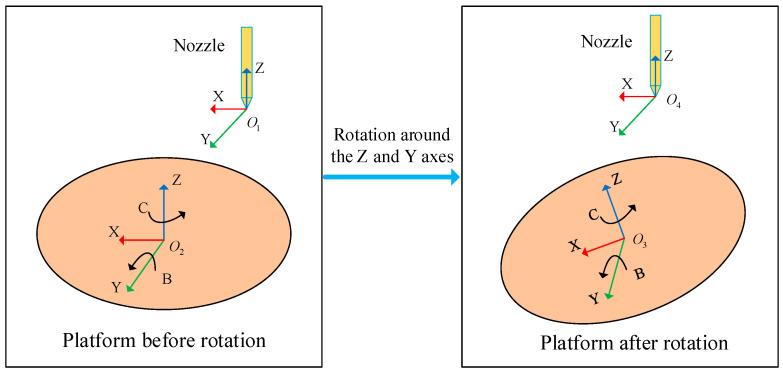
The inverse kinematics analysis of the FAMD.

When performing unsupported printing on an FAMD, it is often necessary to adjust the angle value of the rotation axis (*B*, *C*) to achieve the tilt of the printing platform. The angle value of the rotation axis (*B*, *C*) and the displacement value of the feed axis (*X*, *Y*, *Z*) will affect the position of the printing nozzle, and the displacement of the rotation axis determines the posture of the printing nozzle [26]. To obtain the value of *X*, *Y*, *Z*, *B* and *C*, it is crucial to analyze and solve the inverse kinematics of the FAMD. The coordinate system transformation relationship between the printing platform before and after flipping is shown in Figure 3.

The relationship between the printing nozzle position and the joint variables of the FAMD can be obtained from Equation (6), as shown in Equation (8).

(8) $$\left[\begin{array}{c} x\\ y\\ z \end{array}\right]=\left[\begin{array}{c} XcosBcosC-YsinC+ZsinBcosC\\ XcosBsinC+YcosC+ZsinBsinC\\ ZcosB-XsinB \end{array}\right]$$

By solving Equations (7) and (8), respectively, the values of *X*, *Y*, *Z*, *B* and *C* can be obtained. Each value is shown in Equations (9) to (13).

(9) $$B=\arccos\left(k\right)$$

(10) C=arctan2(j,i)

(11) Y=ycosC−xsinC

(12) $$X=\frac{x+y+Y\left(sinC-cosC\right)}{\left(cosB+sinBtanB\right)\left(cosC-sinC\right)}-\frac{ztanB}{cosB+sinBtanB}$$

(13) $$Z=\frac{z-XsinB}{cosB}$$

## 3. The Method of Support-Free Printing

To realize the support-free printing of the FAMD, it is necessary to perform layering and path planning operations on the model through the host computer to generate G-code files that can be used by the lower computer.

Today’s popular slicing software imports model files that have been converted into STL format and performs layering and path planning operations on them. This results in accuracy problems caused by triangulated models that cannot be avoided. This paper uses Rhino 7.0 (NURBS) software to directly perform layering and path planning operations on the three-dimensional model. Using NURBS can retain the surface accuracy of the CAD model [27]. The surface quality of parts manufactured by plane slicing is affected to a certain extent by the staircase effect, which becomes more pronounced as the profile offset of successive layers increases. Thanks to the application of multi-degree-of-freedom equipment in the field of AM, the FAMD can reduce the adverse effects caused by the staircase effect by changing the direction of the printing nozzle or printing platform [28].

**Figure 4:**
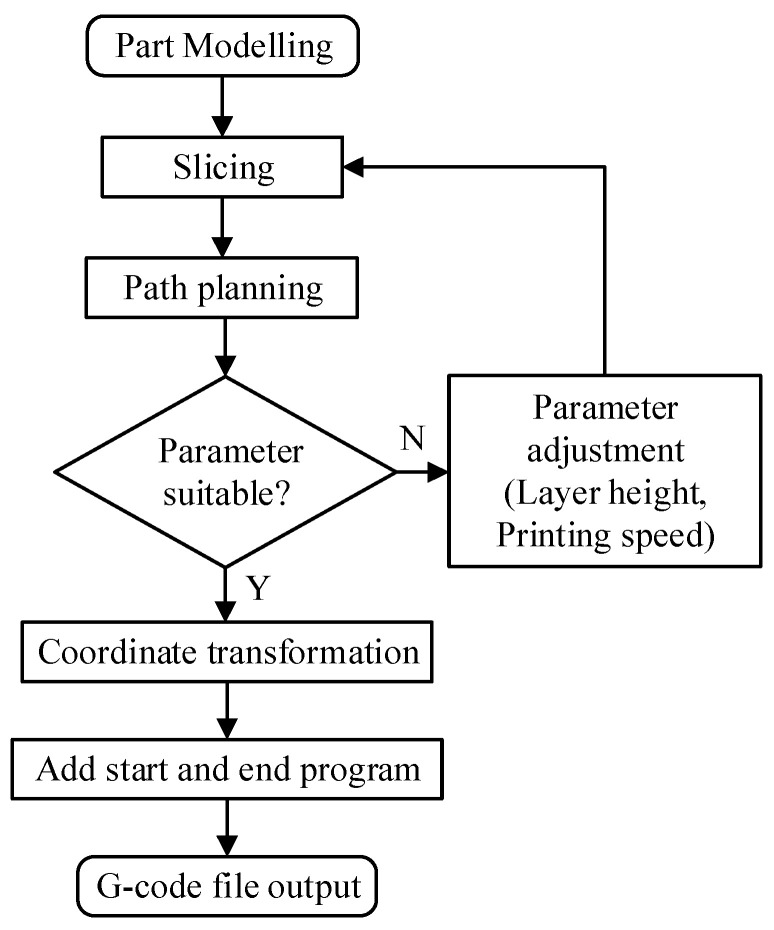
The process of obtaining the G-code file.

In order to achieve the support-free printing of parts, the model to be printed must be converted into a G-code file that can be used by the FAMD. Operations such as model establishment, hierarchical slicing, path planning and coordinate transformation need to be performed. The specific steps for obtaining the G-code file are shown in Figure 4.

### 3.1. Slicing Algorithm Based on Structural Lines

**Figure 5:**
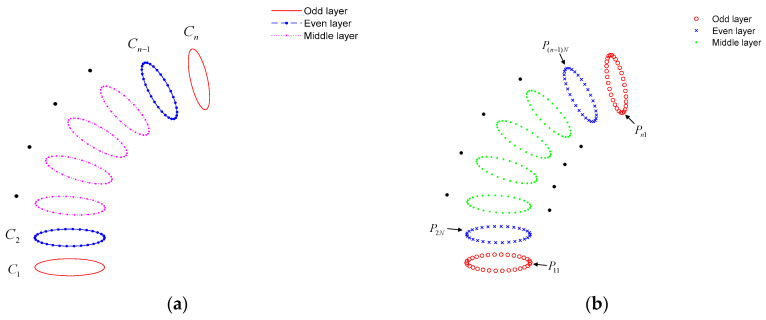
The determination of the outer contours and the starting points of outer contours. (**a**) The determination of the outer contours of odd-numbered layers and even-numbered layers; (**b**) the determination of the starting points of the outer contours of odd-numbered layers and even-numbered layers.

The edge structure line is extracted according to the model characteristics, and n structure points are selected on the structure line according to a certain step size h, and the structure point set is $V=\{V_{1},\;V_{2},\;\ldots ,\;V_{n}\}$. Each structural point is taken as the tangent point, and the tangent line of the tangent point on the structural line is found, and the normal line perpendicular to the tangent line is found. The plane perpendicular to the structural line can be determined through the tangent point and the normal line. By intersecting the vertical plane with the model to be printed, the intersecting plane can be obtained, and the set of intersecting planes is $S=\left\{S_{1},\;S_{2},\;\ldots ,\;S_{n}\right\}$. The edge line segments of the intersecting planes of each layer are extracted and used as the outer contour of each layer after slicing the model. The outer contour set is $C=\left\{C_{1},\;C_{2},\;\ldots ,\;C_{n}\right\}$. The extracted outer contour is shown in Figure 5a. (Only parts of the outer contour are shown).

### 3.2. Continuous Path Planning for Support-Free Printing

Changes in printing parameters will affect the characteristics of the final 3D-printed part [29]. Different printing parameters such as printing temperature, printing direction, printing path and layer thickness will affect the tensile strength, flexural strength, crystallinity and grain size of FDM-printed parts [30]. Therefore, to obtain higher strength parts, a continuous path planning method is adopted. The printing path is divided into an outer contour path and an inner filling path, and the starting points and end points of the outer contour path and the inner filling path are connected alternately to achieve a continuous SFAM path.

#### 3.2.1. Path Planning of Outer Contour

The slicing algorithm based on structural lines can extract the outer contour of each layer of the model, and the set of outer contours is $C=\left\{C_{1},C_{2},\ldots ,C_{n}\right\}$. Each closed outer contour is divided into m segments; that is, each outer contour has m segmentation points. The segmented point set of each outer contour is $P=\left\{P_{1},P_{2},\ldots ,P_{m}\right\}$. The segmented point set of the outer contour of any layer is $P_{i}=\left\{P_{1m},P_{2m},\ldots ,P_{nm}\right\}$, where 1≤i≤n. The specific steps to determine the starting point of the outer contour of each layer are as follows.

Assuming that n is an odd number, the sets of odd-numbered outer contours and even-numbered outer contours are $C_{j}=\left\{C_{1},C_{3},\ldots ,C_{n}\right\}$ and $C_{o}=\left\{C_{2},C_{4},\ldots ,C_{n-1}\right\}$, respectively, as shown in Figure 5a. Additionally, the point set of the outer contours of odd-numbered layers is $P_{j}=\left\{P_{1m},C_{3m},\ldots ,C_{nm}\right\}$, and the point set of the outer contours of even-numbered layers is $P_{o}=\left\{P_{2m},C_{4m},\ldots ,C_{(n-1)m}\right\}$. In order to improve the surface quality of parts, the starting point of the printing path between the outer contours of adjacent layers should not be kept in the same material deposition direction. First of all, the starting point of the even number outer contours is processed, but the odd-numbered outer contour is not. The starting point set of the odd-numbered outer contours is $P_{js}=\left\{P_{11},P_{31},\ldots ,P_{n1}\right\}$, and the original starting point set of the even contour layers is $P_{os}=\left\{P_{21},P_{41},\ldots ,P_{(n-1)1}\right\}$. To achieve the continuity of the printing path, the starting points of the odd-numbered outer contours and the even-numbered outer contours are not on the same side of the model. The middle segmentation points of the even-numbered outer contours are taken as its starting point, as shown in Figure 5b. The specific algorithm is divided into three steps:

1. Take out the segmented point set of the even-numbered outer contours, and the set of the points is $P_{o}=\left\{P_{2m},P_{4m},\ldots ,P_{(n-1)m}\right\}$ (n is an odd number).
2. Find out the intermediate segmentation point N, and its calculation is shown in Equation (14).
  (14) $$N=\left\{\begin{array}{ll} (m/2)+1, & m\;is\;even\\ (m+1)/2, & m\;is\;odd \end{array}\right.$$
3. Determine the starting points of the even-numbered outer contours, and its point set is $P_{os}=\left\{P_{2N},P_{4N},\ldots ,P_{(n-1)N}\right\}$ (n is an odd number).

#### 3.2.2. Path Planning for Interior Infilled Areas

**Figure 6:**
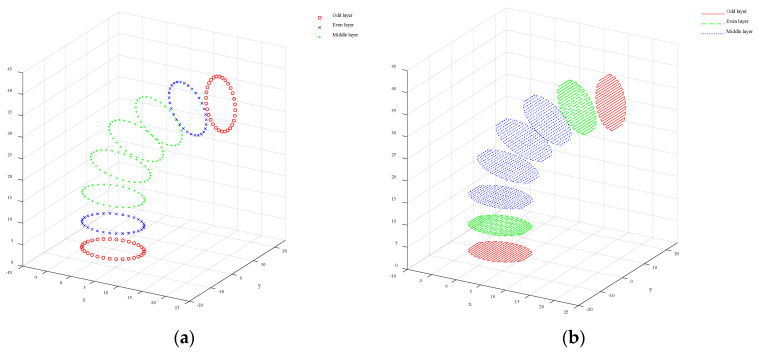
The points of outer contours and the curves within the filled area. (**a**) The points of the outer contours of odd and even layers; (**b**) the Zigzag curves within the filled area.

In order to improve the printing quality and manufacturing efficiency of the part, a parallel line filling path is adopted. According to the intersecting plane set $S=\left\{S_{1},\;S_{2},\;\ldots ,\;S_{n}\right\}$, the boundary rectangles and the longest side of the boundary rectangles can be obtained. Starting from the midpoint of the longest side, the longest sides are divided into F segments, and F + 1 segmentation points are obtained. A straight segment perpendicular to the longest side is made over the segmentation point, and the segments are projected onto the intersection plane to obtain the inner filling segments of each layer of the model. Commonly used 3D printing processes are divided into path-based continuous filling and on-demand array filling according to filling methods. In order to achieve continuous printing in the inner filling area and reduce the idle stroke movement of the nozzle, the Zigzag filling method is used to plan the path of the inner filling part, as shown in Figure 6.

To obtain a continuous five-degree-of-freedom AM path, it is necessary to merge the outer contour path of each layer with the inner filling path. The steps are as follows: starting from the starting point of the outer contour of the n layer and ending at the end point, a continuous closed outer contour curve is formed; starting from the starting point of the inner fill path in the n layer (i.e., the end point of the outer contour path of the n layer) and ending at the end point of the inner fill path, a continuous inner fill path curve is formed; using the end point of the filling path in the n layer as the previous path point of the starting point of the outer contour of the n+1 layer and repeating the operation until the last layer to form a continuous five-degree-of-freedom printing path. Finally, coordinate transformation is performed on all path points, the start and end codes are added, and the G-code file is exported to prepare for the printing experiment.

## 4. Experiment and Discussion

**Figure 7:**
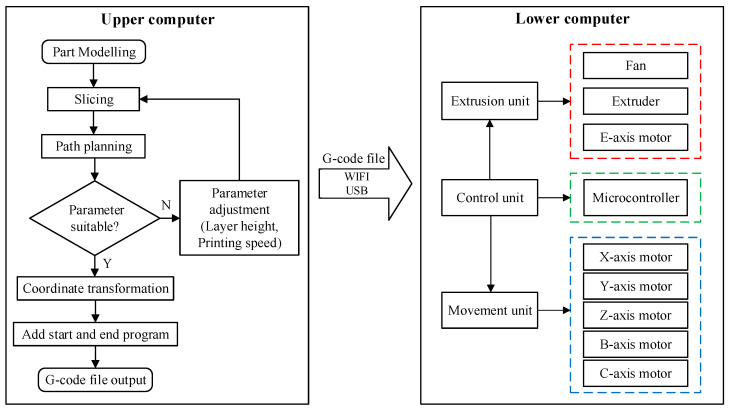
The system of SFAM.

**Figure 8:**
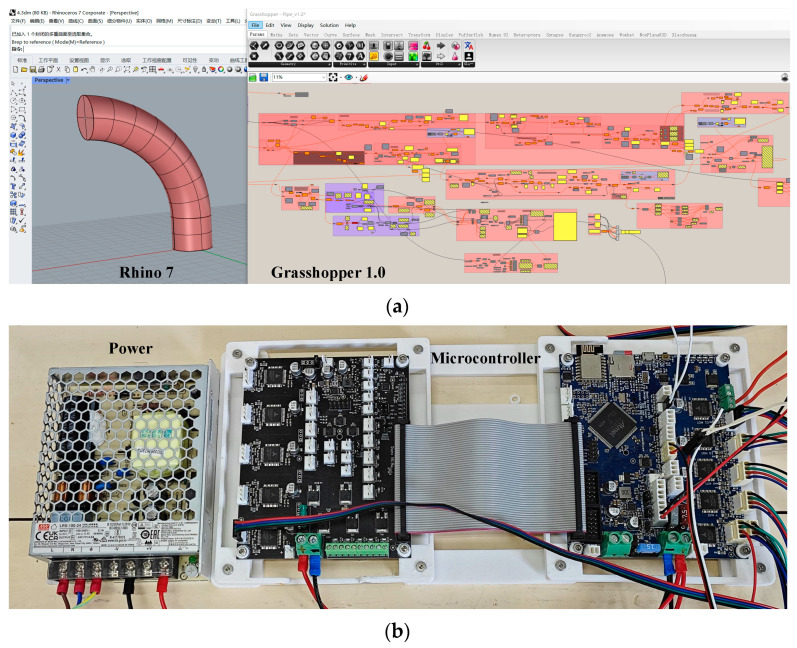
The upper computer and the lower computers. (**a**) The upper computer used to complete the operation of slicing and path planning; (**b**) the lower computer which keeps the movement unit and the extrusion unit under control to print the parts correctly.

The five-degree-of-freedom AM system consists of the upper computer and the lower computer to achieve different functions, as shown in Figure 7. The slicing and path planning program is written in the Grasshopper 1.0 software of the upper computer, which generates a series of path points, and the three-axis coordinate points are converted into five-axis coordinate points suitable for FAMD. Additionally, the start (pre-processing) and end instructions, the printing speed parameters and extrusion volume are added so that the G-code file can be generated by exporting all data, as shown in Figure 8a. The upper computer exports the G-code file and uploads it to the lower computer which receives and reads the G-code file and keeps the movement unit (marked in blue in Figure 7) and the extrusion unit (marked in red in Figure 7) under control to print the parts correctly, as shown in Figure 8b.

### 4.1. Comparison of Path Planning Algorithms

When a TTAM device manufactures parts with overhanging features, it is necessary to print the support part of the same layer while printing the part. However, the printing path of the support structures and parts is often discontinuous so that it will cause the printing nozzle to have a large amount of idle motion during the manufacturing process, which greatly reduces manufacturing efficiency and increases manufacturing costs.

A support-free continuous manufacturing path is used in the SFAM method in this paper. There is no need to add extra structures when printing overhang feature models, and support-free printing greatly reduces the use of printing materials. Between adjacent layers, the AM path is continuous, which can reduce the idle motion of the printing nozzle and improve manufacturing efficiency.

### 4.2. Comparison of Manufacturing Costs of Parts

**Figure 9:**
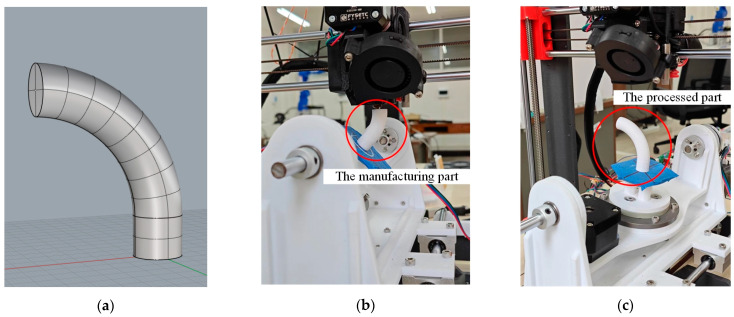
The complete manufacturing process of the part with a circular cross-section. (**a**) The model of the part with a circular cross-section; (**b**) the process of manufacturing the part (marked with a red circle); (**c**) the processed part (marked with a red circle).

The model shown in Figure 9a was manufactured by the TTAM method of three-axis slicing software Simplify 3D 4.0 and the SFAM method of this study. The diameter and height of the model are 12 mm and 46 mm, respectively. The material used for printing is PLA, and the nozzle diameter is 0.4 mm. By comparing the printing time and filament usage of parts manufactured by the two methods, the surface quality of the parts was observed, and the surface roughness of the parts was measured.

As shown in Figure 9a, when the overhang angle of the printed model exceeds more than 45°, the TTAM device requires adding support structures to assist in printing. However, the FAMD does not require the addition of support structures at all when printing parts with overhang features, as shown in Figure 9b. The larger the overhang angle of the model, the more support part is added when printing the part using the TTAM method, and the longer it takes to manufacture.

**Figure 10:**
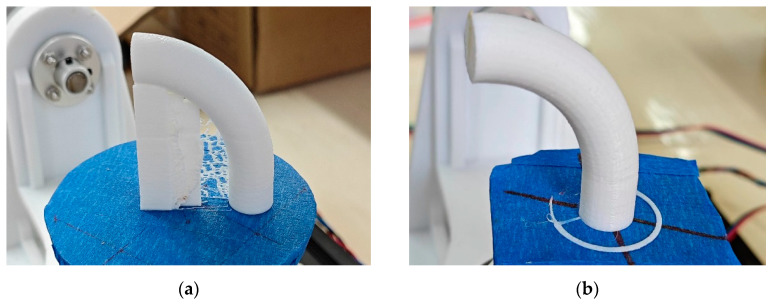
Comparison of parts printed by two different methods. (**a**) Part with circle cross-section manufactured by TTAM method; (**b**) part with circle cross-section manufactured by SFAM method.

The parts shown in Figure 10 are printed by the TTAM method and the SFAM method, respectively. The printing time and filament usage of parts manufactured by the two different methods are listed in Table 1. It can be seen that compared with TTAM methods, the time required to print parts using the SFAM method is reduced by 23.58%, and the usage length of the filament is reduced by 33.06%, which means that printing efficiency is significantly improved, and the printing cost is also significantly reduced.

### 4.3. Comparison of Surface Quality of Parts

**Figure 11:**
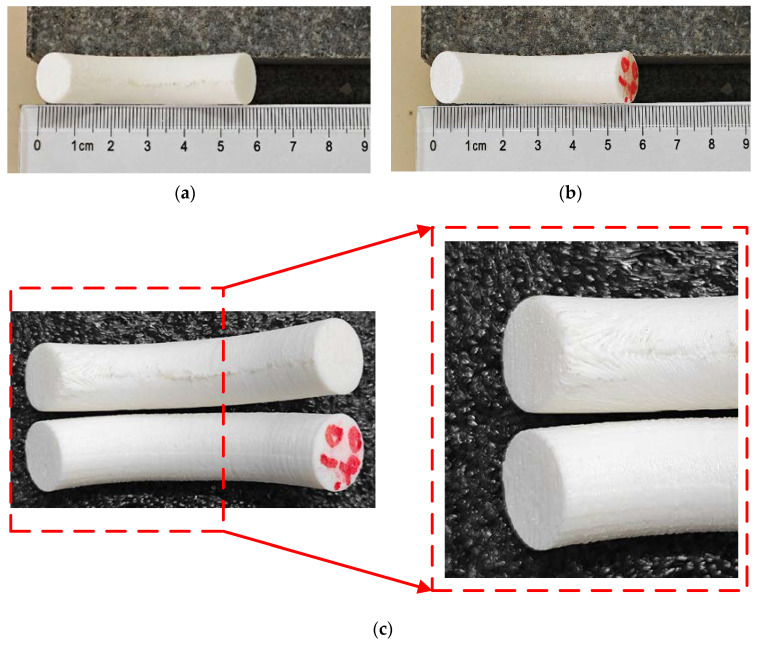
A comparison of the side surface of the processed parts. (**a**) The sideThe specific algorithm is divided into three surface of the part with a circle cross-section manufactured by the TTAM method; (**b**) the side surface of the part with a circle cross-section manufactured by the SFAM method; (**c**) partial enlarged detail of the side surface on the top of two parts.

As shown in Figure 11a,b, the part printed by the TTAM method needed to remove the support structures, resulting in uneven spots on the contact surface (side surface) between the part and the support structures, which makes its surface much less smooth than the side surface of the part printed using the SFAM method (marked in red on the bottom).

Figure 11c shows a comparison of the top side surface of two parts. The quality of the top side surface of the part manufactured by the SFAM method is better. It can be clearly seen that there are unbonded areas on the side surface of the part printed by the TTAM method, and the stair effect is serious. This is due to the fact that the top of the part has the largest overhang angle and the largest contact area with the support structures, and the post-processing operation causes a serious degradation in the quality of the contact surface of the part. In contrast, using the SFAM method to manufacture the overhang part can avoid a series of problems caused by support structures.

**Figure 12:**
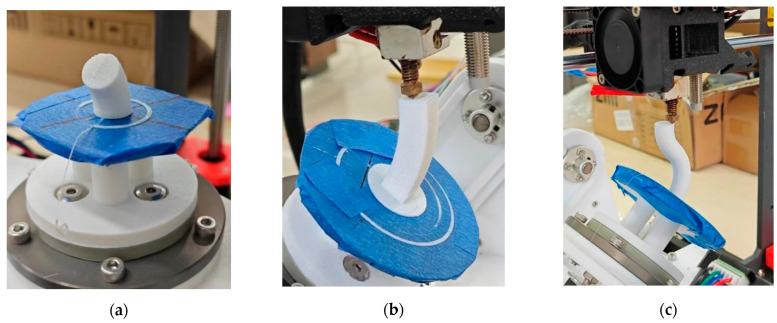
Manufacturing of parts with other shapes. (**a**) Part with circular cross-section. (**b**) Manufacturing process for parts with rectangular cross-sections. (**c**) Manufacturing process for S-shaped part.

Figure 12 shows the parts of different shapes printed by the SFAM method. The finished parts of the circular cross-section model, rectangular cross-section model and “S” model are shown in Figure 12a–c, respectively.

**Figure 13:**
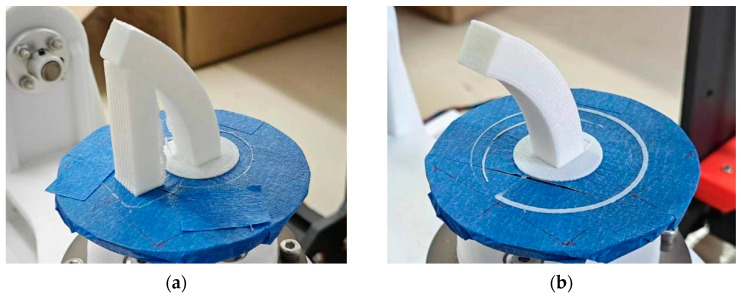
Comparison of rectangular cross-section models manufactured by two different methods. (**a**) Part with rectangular cross-section manufactured by TTAM method; (**b**) part with rectangular cross-section manufactured by SFAM method.

**Figure 14:**
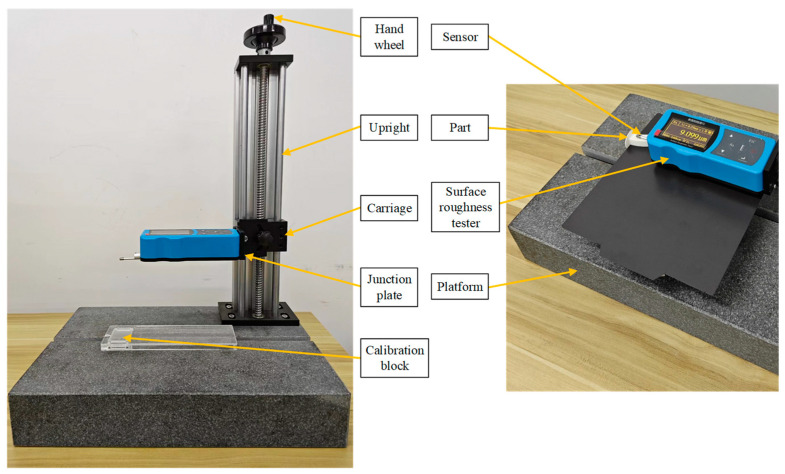
The surface roughness tester and the process of testing the roughness of the part.

**Figure 15:**
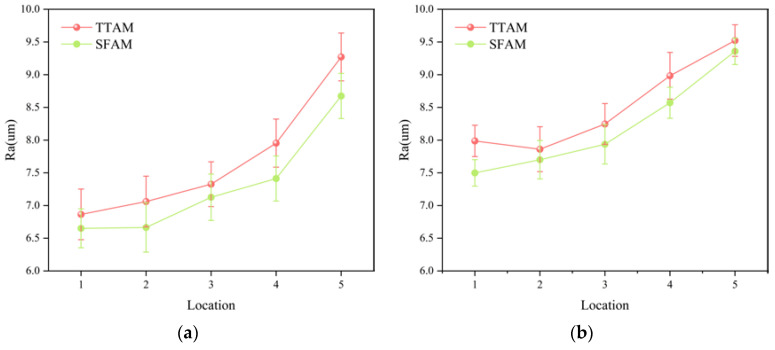
Comparison of side surface roughness of rectangular cross-section parts. (**a**) Comparison of surface roughness on left side; (**b**) comparison of surface roughness on right side.

As shown in Figure 13, the parts of the rectangular cross-section model were printed by the TTAM method and the SFAM method, respectively. The surface roughness tester shown in Figure 14 was used to detect the roughness of side surfaces (left side and right side) of the parts, respectively, and the detection results are shown in Figure 15. As can be seen from Figure 15, compared with parts manufactured by the TTAM method, the side surface roughness of the part manufactured by the SFAM method is lower, which indicates that its surface quality is better.

## 5. Conclusions

In order to solve the problem that TTAM needs to add support structures when printing overhanging parts, an SFAM method is proposed, which adopts a continuous path planning algorithm. Using this method to manufacture parts with overhanging structures can reduce the idle motion of the printing nozzle and enables the support-free printing of the device. The printing experiments of parts were conducted on an FAMD. The experimental results show the following:

(1) Compared with the TTAM method, the time to manufacture parts using the SFAM method is reduced by 23.58%, and material is saved by 33.06%, which greatly reduces manufacturing costs and improves manufacturing efficiency.
(2) Compared with the TTAM method, there is no need to generate support structures when printing parts with overhang structures using the SFAM method, which avoids accuracy problems caused by removing support parts and makes the surface of parts smoother and has a better surface quality.

When the model has complex shape features such as multi-branch structure and large curvature, it cannot be printed normally. The more branching structures the model has, the more difficult the parts are to manufacture. Combined with the currently designed SFAM method, more time and energy need to be invested in the future to complete the support-free manufacturing of parts with complex structural shapes and large surface curvatures.

## Figures and Tables

**Table 1:** A comparison of printing time and filament length for parts manufactured by the TTAM method and the SFAM method.

Method	Layer Thickness (mm)	Extrusion Width (mm)	Printing Speed (mm/min)	Printing Time (min)	Filament Length (mm)
TTAM	0.20	0.60	720.00	106.00	2904.70
SFAM	0.20	0.60	720.00	81.00	1944.30
Result				−23.58%	−33.06%

## Data Availability

The original contributions presented in the study are included in the article, further inquiries can be directed to the corresponding authors.
